# Prognostic significance of supradiaphragmatic lymph node metastasis detected by ^18^F-FDG PET/CT in advanced epithelial ovarian cancer

**DOI:** 10.1186/s12885-018-5067-1

**Published:** 2018-11-26

**Authors:** In Ok Lee, Jung-Yun Lee, Hyun Jeong Kim, Eun Ji Nam, Sunghoon Kim, Sang Wun Kim, Chang Young Lee, Won Jun Kang, Young Tae Kim

**Affiliations:** 1 Department of Obstetrics and Gynecology, National Health Insurance Service Ilsan Hospital, Goyang-si, Gyeonggi-do South Korea; 2 Department of Obstetrics and Gynecology, Institute of Women’s Life Medical Science, Yonsei University College of Medicine, 50-1 Yonsei-ro, Seodaemun-gu, Seoul, 03722 South Korea; 3 Department of Nuclear Medicine, Yonsei University College of Medicine, 50-1 Yonsei-ro, Seodaemun-gu, Seoul, 03722 South Korea; 4 Department of Chest Surgery, Yonsei University College of Medicine, Seoul, South Korea

**Keywords:** Ovarian cancer, Ovarian neoplasm, Supradiaphragmatic lymph node, PET/CT, Survival, Recurrence pattern

## Abstract

**Background:**

Supradiaphragmatic lymph node metastases (SdLNM) are frequently identified using ^18^F-FDG positron emission tomography/computed tomography (PET/CT) in advanced epithelial ovarian cancers (AEOC). This study aimed to determine the prognostic significance of SdLNM detected by PET/CT in patients with AEOC.

**Methods:**

Medical records of patients diagnosed with AEOC were retrospectively registered from January 2009 to July 2015. Patients were categorized according to PET/CT stage: PET/CT stage III, PET/CT stage IV with SdLNM, and PET/CT stage IV with other metastases. Clinicopathologic characteristics, recurrence patterns, survival outcomes were compared according to PET/CT stage. Anatomical distribution of SdLNM and effect of thoracic debulking surgery were estimated.

**Results:**

A total of 295 patients were identified, including 176 patients who underwent primary debulking surgeries (PDS). Progression-free (*P* = 0.671) and overall (*P* = 0.525) survival did not differ significantly between patients with PET/CT IV with SdLNM and PET/CT IV with other metastases; however, patients with PET/CT IV with SdLNM had significantly poorer progression-free (*P* < 0.001) and overall (*P* = 0.016) survival than those with PET/CT stage III. Recurrence patterns were similar in all groups; intraperitoneal metastasis was the most common (78.8%) and thoracic recurrence alone accounted for less than 10%. Debulking of SdLNM lesions did not improve progression-free survival (*P* = 0.425) or overall survival (*P* = 0.465) of patients with AEOC.

**Conclusions:**

SdLNM detected using preoperative PET/CT are a negative prognostic factor in AEOC. Resection of suspicious SdLNM may not have effect to survival of patients with AEOC.

**Electronic supplementary material:**

The online version of this article (10.1186/s12885-018-5067-1) contains supplementary material, which is available to authorized users.

## Background

Complete tumor resection is important in patients with advanced epithelial ovarian cancer (AEOC). Although optimal debulking surgery leaving < 1 cm of residual tumor is known to improve the overall survival of patients with AEOC [1, 2], complete debulking surgery leaving no macroscopic disease can maximize the survival benefit [3, 4]. Most studies describing optimal or no gross residual disease have only evaluated intraperitoneal disease; only a few studies have examined extraperitoneal disease so far.

After introducing of ^18^F-FDG positron emission tomography/computed tomography (PET/CT) scanning for preoperative evaluation, extraperitoneal disease has raised further questions about management of AEOC. PET/CT could better discriminate malignancy from benign ovarian tumor, compared to other preoperative imaging modalities such as ultrasonography, abdominopelvic computed tomography (CT) or magnetic resonance image (MRI) [5]. Distant metastases including nodal involvement could be predicted more accurately by preoperative PET/CT than CT [6]. Nonetheless in an era of extensive preoperative imaging modalities, management of AEOC with supradiaphragmatic lymph node metastasis (SdLNM) following an initial PET/CT remains uncertain [7].

The prognosis of pathologically confirmed SdLNM has not been clarified for AEOC. There has been little research of the prognostic impact of SdLNM in AEOC. Fruscio et al. found that PET/CT led to an upstaging of about one fourth of ovarian cancer patients from stage III to IV after SdLNM detection [8]; however, they concluded that the prognosis of patients with stage IV metastases detected by PET/CT was similar to that of those at stage IIIC. In another study, recurrence pattern of stage IV ovarian cancer patients was evaluated to determine the benefit of thoracic cytoreduction [9]; however, since thorax-only recurrence was very rare, the authors assumed that thoracic debulking would not change the course of stage IV ovarian cancer. None of the previous studies reported any association between surgical resection and pathology of SdLNM detected using PET/CT. Therefore, this study aimed to determine the prognostic significance of SdLNM detected using PET/CT in patients with AEOC.

## Methods

### Study population

We retrospectively reviewed the medical records of patients diagnosed with ovarian cancer after surgical management of pelvic mass or peritoneal disease, between January 2009 and July 2015. Patients with available preoperative PET/CT scan images, at stage IIIB to IVB after surgical staging, based on the 2014 International Federation of Obstetrics and Gynecology (FIGO) ovarian cancer staging system, were isolated. Clinico-pathologic factors of those patients, such as age, initial serum cancer antigen 125 (CA-125) levels, histology of the epithelial ovarian malignancy, and histologic grades were collected and analyzed. This study was approved by the institutional review board of Yonsei University College of Medicine.

Regarding preoperative imaging modalities for suspicious AEOC, patients had CT or MRI scans and PET/CT. Based on preoperative imaging stage, medically inoperable patients underwent neoadjuvant chemotherapy (NAC) followed by interval debulking surgery (IDS). Patients who were scored over 8 based on the laparoscopic prediction model [10] also underwent NAC followed by IDS. Remaining patients underwent primary debulking surgery (PDS). The patients in the PDS group were further classified based on their PET/CT imaging stages. The PET/CT stage III group included patients who had only intraperitoneal disease without other organ involvement (PET/CT III). The PET/CT stage IV group was divided into two sub-groups: one group included patients who had ^18^F-FDG uptake only in supradiaphragmatic lymph nodes (SdLN), without evidence of lesions outside the abdomen or of other organ involvement (PET/CT IV SdLNM), and the other group included patients with distant organ involvement, such as the liver, spleen, and lung parenchyma, regardless of SdLN involvement (PET/CT IV other).

The extent of surgical resection was based on the clinician’s discretion, per the universal goal for AEOC to achieve complete resection of the disease. Debulking surgeries including not only intra-peritoneal procedures, but also supradiaphragmatic procedures were investigated. Intra-peritoneal procedures included hysterectomy, bilateral salpingo-oophorectomy, omentectomy, pelvic and/or para-aortic lymphadenectomies, peritonectomy for peritoneal lesions, various lower gastro-intestinal system surgeries, liver resection, and splenectomy for suspicious lesions. Supradiaphragmatic procedures consisted of video-assisted thoracoscopic surgery (VATS) for mediastinal lesions, either biopsy or excision of the supraclavicular fossa lymph node (SCFLN), and either biopsy or excision of the axillary lymph nodes (AxLN). The residuals of disease in intraperitoneal cavity after all the procedures were investigated. Taxane-platinum combination chemotherapy was done for all patients in this study, which made more homogenous population for analysis. Median cycle number of the chemotherapy after PDS was 6 cycles in this study. Finally, recurrences and patient survival rates were collected.

### PET/CT technique

Patients were instructed to fast for over 8 h before PET image acquisition. Blood glucose concentrations were confirmed to be lower than 140 mg/dL; 5.5 MBq of ^18^F-FDG per kg body weight was injected intravenously, with patients lying comfortably for an uptake period of 60 min. Integrated FDG-PET/CT was performed using a dedicated PET/CT scanner (Discovery STE; GE Healthcare, Milwaukee, WI). Unenhanced CT scan was performed from the vertex of the skull to the mid-thigh using the following parameters: 120 kVp, 30 mA, 0.8-s rotation time, 3.75 mm helical thickness, 27 mm per rotation (speed), 2.5 mm scan reconstruction, with a reconstruction index of 1.25 mm, 15.7 cm field of view, and a 512 × 512 matrix. A PET scan was then acquired from the cerebellum to the proximal thigh with an acquisition time of 3 min per bed position in the three-dimensional mode. PET data were reconstructed iteratively using an ordered-subset expectation maximization algorithm.

### Image analysis

All ^18^F-FDG PET/CT images were independently reviewed visually by two nuclear medicine physicians. One nuclear medicine physician has 15 years of expertise and the other has 7 years of it. Each region with ^18^F-FDG uptake higher than the background was considered significant after exclusion of physiologic uptake. For the quantitative analysis, the maximum standardized uptake (SUVmax) values were measured by drawing a circular region of interest (ROI) at the site of the maximum ^18^F-FDG uptake on transaxial PET images. The SUVmax of the ROI was calculated as follows: [decay-corrected activity (MBq) per tissue volume (mL)]/ [injected ^18^F-FDG dose (MBq) per body mass (g)]. The sizes of the SdLNs were measured on CT images and recorded for data analysis.

**Fig. 1:**
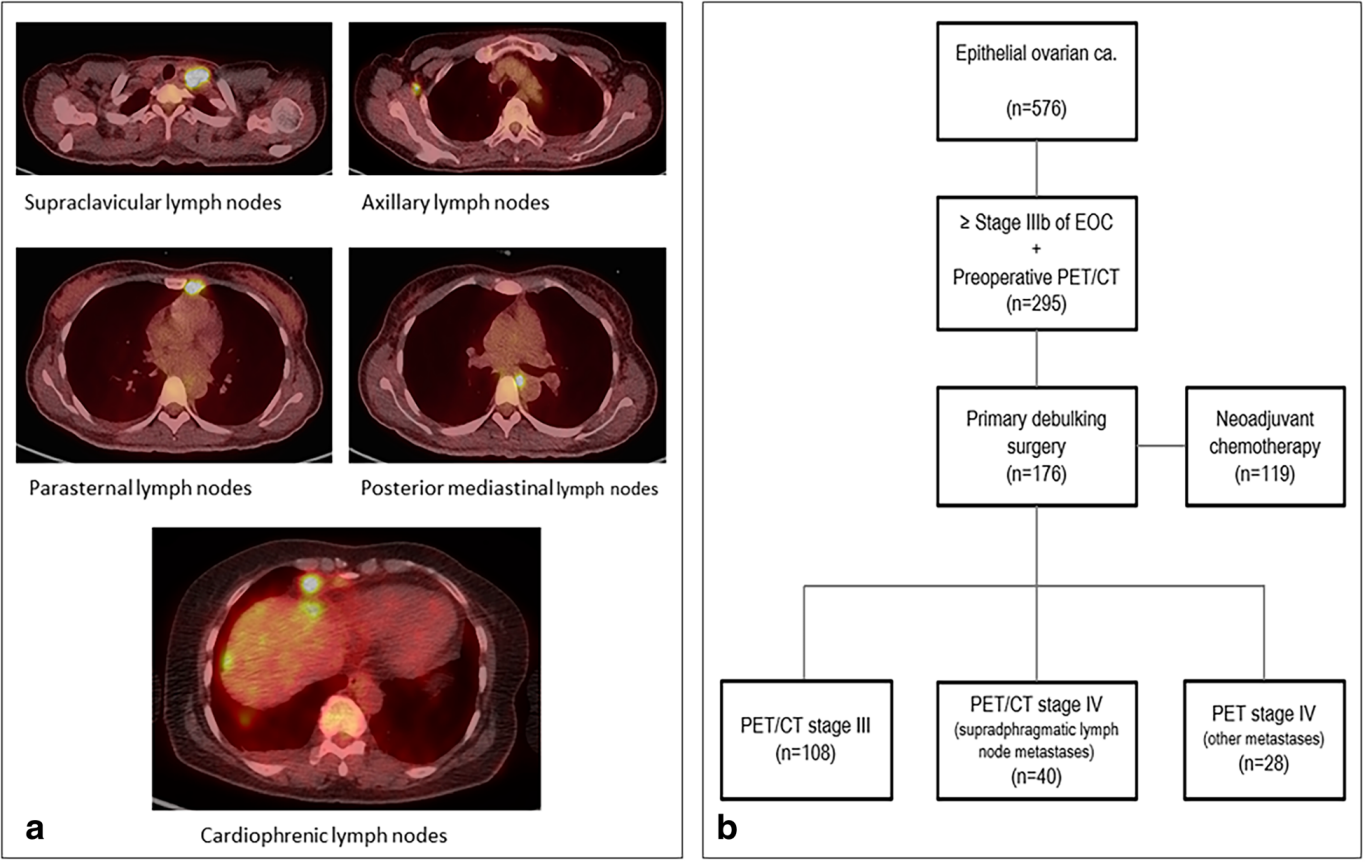
**a** Five groups of supradiaphragmatic lymph node metastasis on PET/CT, **b** flow diagram

The SdLNs consisted of five subdivisions. The lymph nodes in the anterior mediastinal space were divided into two groups: cardiophrenic lymph nodes (CPLN) in the lower space and parasternal lymph nodes in the upper space (Fig. 1a). Medial and posterior mediastinal lymph nodes were counted as one group. In addition, dimensions of the SCFLN, and AxLN and their SUVmax were measured.

### Statistical analysis

To compare groups, a two-tailed Student t-test and Fischer’s exact test were used. Kaplan-Meier estimates and log-rank tests were performed to analyze survival. Hazard ratios (HRs) for survival were estimated with a Cox proportional hazards model. SPSS version 23.0 (IBM Corp, Armonk, NY, U.S.A.) was used to analyze the data.

## Results

From January 2009 to July 2015, 576 patients underwent surgery for and were diagnosed with primary epithelial ovarian cancer at our institution (Fig. 1b). Among them, 336 patients had staged IIIB to IVB primary epithelial ovarian cancer. Patients who did not undergo initial PET/CT evaluations were excluded. Finally, data from 295 patients were analyzed, of which 176 patients underwent PDS and 119 patients underwent NAC followed by IDS. Among the patients who underwent PDS, 108(55.9%) belonged to the PET/CT III group. Forty patients (24.4%) had only showed SdLNM on preoperative PET/CT scans, and 28 patients (19.7%) showed evident involvement of other organ on the PET/CT scans. No statistically significant difference was noted in preoperative serum CA-125 levels among the groups (*p* = 0.13). Similarly, other clinicopathologic characteristics including residual disease after PDS (*p* = 0.405) were not significantly different among the three groups (Table 1).

**Table 1:** Patient characteristics

	total n	%	PET_CT stage III (n, %)		PET_CT stage IV by SdLNM (n, %)		PET_CT stage IV by other (n, %)		*p*-value
median Age (years, IQR)	176	100	108	55.9	40	22.4	28	19.7	0.627
	55 (48–63)		54 (47–63)		55 (49–63)		55 (48–60)		
median initial CA-125 (U/mL, IQR)	848.05 (334.97–2387.32)		659.35 (296.20–2446.90)		1111.80 (712.0–2483.15)		1048.15 (148.45–2111.65)		0.130
FIGO stage									
IIIB	11	6.3	8	7.4	3	7.5	0	0.0	
IIIC	100	56.8	76	70.4	13	32.5	11	39.3	
IVA	4	2.3	3	2.8	1	2.5	0	0.0	
IVB	61	34.7	21	19.4	23	57.5	17	60.7	0.502
Histology									
high grade serous	136	77.3	82	75.9	34	85.0	20	71.4	
others	40	22.7	26	24.1	6	15.0	8	28.6	0.423
Grade									
1	14	8.0	10	9.3	2	5.0	2	7.1	
2	59	33.5	38	35.2	13	32.5	8	28.6	
3	88	50.0	50	46.3	23	57.5	15	53.6	0.122
unknown	15	8.5		0.0		0.0		0.0	
Residual disease (NGR vs other)									
NGR	38	25.9	23	26.1	10	27.8	5	21.7	
any residuals	109	74.1	65	72.2	26	72.2	18	78.3	0.804
unknown	29		20		4		5		

*NGR* No gross residual disease

Among the patients with PDS, 50 (28.4%) had ^18^F-FDG uptake in the SdLNs on preoperative PET/CT. The most-frequent SdLNM detected by PET/CT was observed in the SCFLN (42%) (Table 2). Median size of SdLNM was 7.65 mm. There were 27 patients (54.0%) who underwent resection of thoracic lesions for SdLNM identification, of which two patients underwent only fine-needle biopsy for suspicious SCFLN to confirm metastasis. For the remaining 25 patients, a surgeon from Department of Chest Surgery performed either VATS or thorough excision of lesions during their PDS. Among the 25 patients who underwent thoracic debulking surgery, 21 patients (77.8%) presented with pathologic SdLNM and four patients had only inflammatory changes.

**Table 2:** Incidence of supradiaphragmatic lymph node metastases (SdLNM) in PDS group

SdLNM location	n	%	median size (mm, range)	median SUVmax (range)	pathologic confirmed metastasis (N of pathologic positive /N of excision or biopsy done)
Cardiophrenic	15	30.0	7.65 (3.9–13.0)	2.6 (1.4–14.1)	8/10
Parasternal	15	30.0	6.6 (4.9–15.7)	2.4 (1.2–10.7)	7/8
middle/posterior mediastinal	7	14.0	10 (3.5–17.6)	4.2 (2.3–9.4)	1/2
Axillary	10	20.0	9 (4.4–19.3)	2.6 (1.1–8.1)	3/3
Supraclavicular or Neck node	21	42.0	9.1 (3.5–24.9)	3.2 (1.2–14.7)	5/6

Of the entire study cohort, including the NAC and PDS groups, 151 patients (51.2%) experienced recurrence. Intraperitoneal recurrence was the most-common type of recurrence (78.8%), and < 10% of the patients showed recurrence in thoracic space only (Table 3). The recurrence patterns were similar among the three groups, regardless of imaging stage. Three of the patients who experienced recurrence only in thoracic space died (overall survival rate 72.7%); on the other hand, 43 patients who experienced recurrence only in the intraperitoneal space died (overall survival rate 63.2%). However, the survival outcomes did not show a statistical significance (*p* = 0.634).

**Table 3:** Recurrence pattern depends on primary disease extent by PET_CT

	Total		PET_CT stage III		PET_CT stage IV by SdLNM		PET_CT stage IV by other	
Numbers of recurrence	151		75		41		35	
Recurrent rate (%)	51.2		45.5		56.9		60.3	
Location of recurrence	N	%						
Thoracic space	11	7.3	6	8.0	2	4.9	3	8.6
Intraperitoneal space	119	78.8	60	80.0	35	85.4	24	68.6
Thoracic + Intraperitoneal space	12	7.9	5	6.7	1	2.4	6	17.1
Pleural effusion only	5	3.3	2	2.7	2	4.9	1	2.9
other (brain, bone, skin)	3	2.0	1	1.3	1	2.4	1	2.9
unknown (f/u loss)	1	0.7	1	1.3	0	0.0	0	0

**Fig. 2:**
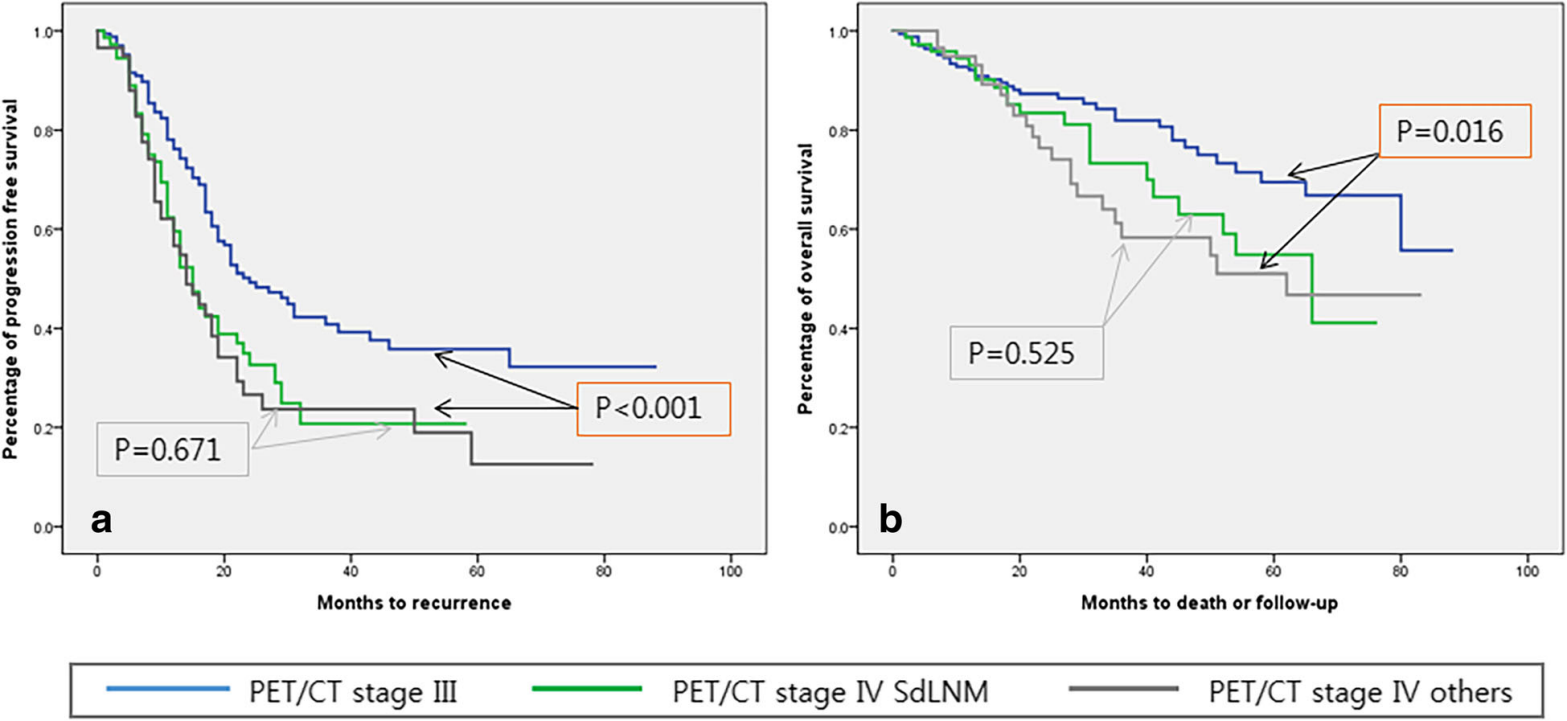
Kaplan-Meier curves of progression-free survival (**a**) and overall survival (**b**) according to PET/CT stages

**Fig. 3:**
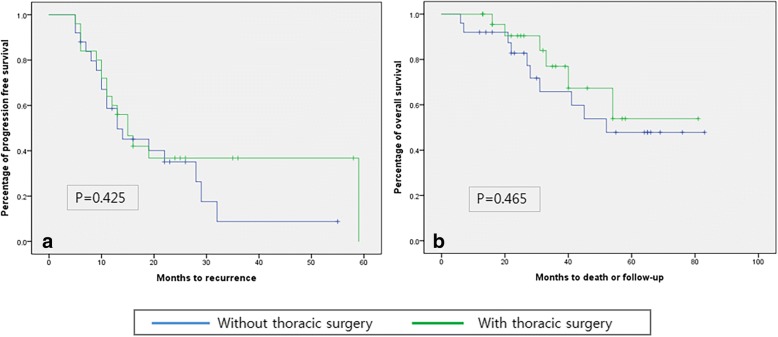
Kaplan-Meier curves of progression-free survival (**a**) and overall survival (**b**) according to thoracic debulking

Both progression-free (*P* = 0.671) and overall (*P* = 0.525) survival did not differ significantly between patients with PET/CT IV with SdLNM and those with PET/CT IV with other metastases; however, those with PET/CT IV with SdLNM had significantly poorer progression-free (median PFS: 14 months, *P* < 0.001) and overall (median OS: 31.5 months *P* = 0.016) survival than those with PET/CT stage III (median PFS: 18 months, median OS: 37.5 months) (Fig. 2). Patients who had thoracic debulking surgery during PDS did not show a progression-free-survival (*P* = 0.425) or overall survival (*P* = 0.465) benefit compared to patients who did not undergo a supradiaphragmatic debulking procedure (Fig. 3).

**Fig. 4:**
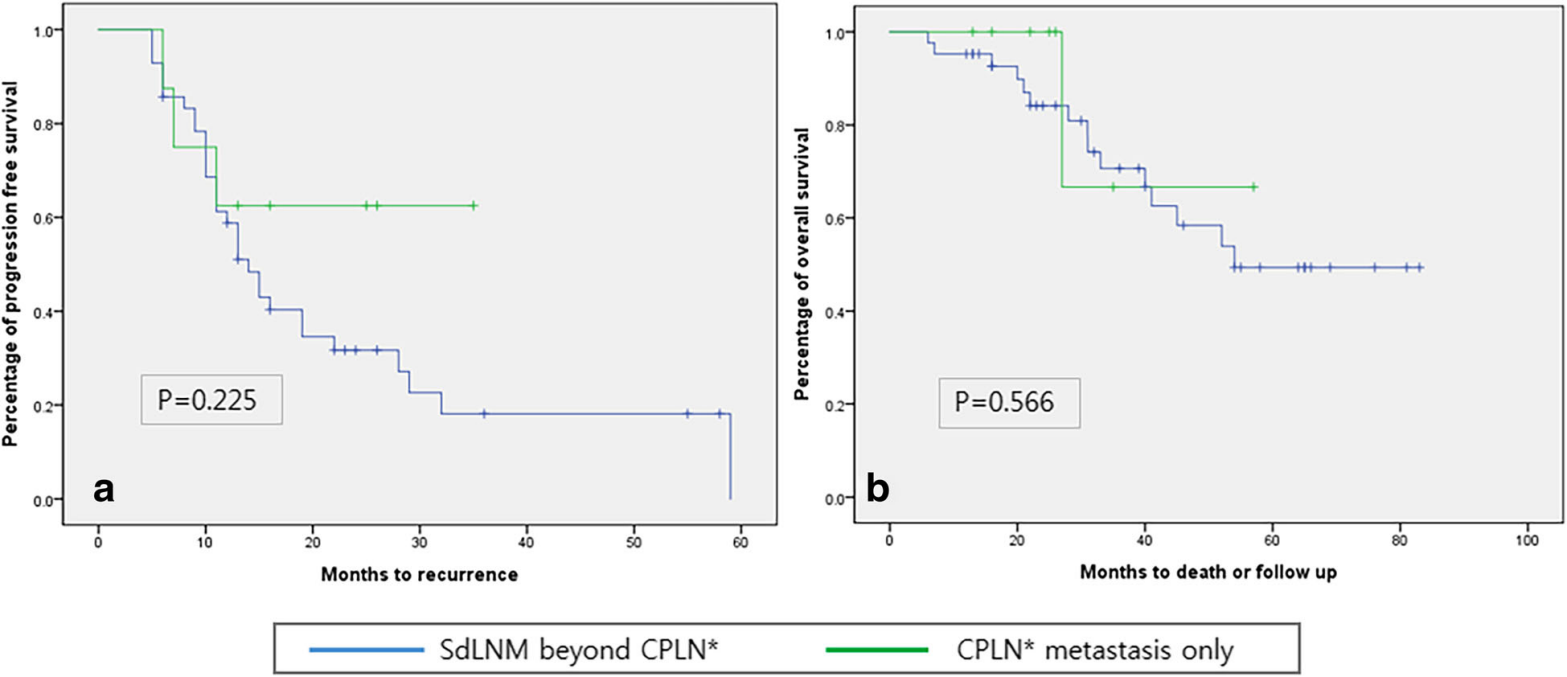
Kaplan-Meier curves of progression-free survival (**a**) and overall survival (**b**) according to location of supradiaphragmatic lymph node metastases (SdLNM)

There were 8 patients who had only CPLN metastases without other extraperitoneal metastases in PET/CT, and 42 patients had SdLNM. Although the group of the patients with CPLN metastases only experienced lower rate of recurrence and death, PFS and OS of the group did not show statistical differences (*p* = 0.225, 0.566, respectively) (Fig. 4) compared to the group of the patients having SdLNM.

No residual disease after PDS (HR = 0.52, 95% CI = 0.29–0.93, *p* = 0.004) and initial PET/CT stage IV including both SdLNM and other metastases (HR = 1.61, 95% CI = 1.13–2.29, *p* = 0.008) were the main contributing factors for progression-free survival of the patients after multivariate analysis (Additional file 1: Table S1).

## Discussion

The present study showed that SdLNM detected by preoperative PET/CT is a significant negative prognostic factor in AEOC. Patients in the PET/CT IV group with SdLNM and the PET/CT IV group with other metastases showed similar survival outcomes. Moreover, resection of suspicious lymph nodes may not have affected survival of patients with SdLNM detected by PET/CT. To the best of our knowledge, this is the first study to pathologically and surgically validate the results of PET/CT for SdLNM detection and to determine their prognostic impact in patients with AEOC.

The definition and location of the SdLN has been confused when describing metastatic disease. These lymph nodes, located beneath the sternum and immediately above the heart, have been named differently as paracardiac, cardiophrenic, or supradiaphragmatic lymph nodes, depending on the study [11–13]. The former two lymph nodes are actually chest wall (parietal) lymph nodes, which are not usually involved in lung or heart diseases that consist of major thoracic organ problem [14]. The chest wall lymph nodes are believed to be afferents of whole abdominal cavity circulation [15].

Compared to CT alone, PET/CTs increase the probability of SdLNM discovery during preoperative imaging. The advantage of whole-body PET/CT is that it allows a surgeon to discover suspicious extra- and intra-peritoneal metastases. Farmakis et al. found that PET/CT was more accurate than CT alone for identifying CPLNs [16]. Similarly, Fruscio et al. also concluded that PET/CT was a better methodology than CT for detecting distant metastases in AEOC [8]: PET/CT accurately upstaged 26% of the patients from FIGO stage III to IV during preoperative PET/CT imaging. In addition, Hynninen et al. reported that preoperative PET/CT detected SdLNM in 67% of patients with advanced ovarian cancer, which is almost double the detection rate with CT alone [17].

Suspicious supradiaphragmatic lymph nodes on preoperative image have been regarded as a negative predictor of AEOC. Holloway et al. reported a relationship between the extent of peritoneal metastasis and enlargement of the paracardiac lymph nodes [11]. Kolev et al. investigated supradiaphragmatic lymphadenopathy detected on preoperative CT in patients with primary ovarian cancer and found that patient survival decreases with SdLN enlargement [12]. Raban et al. showed similar results for the treatment of stage IIIC ovarian cancer: Patients showing paracardiac lymph node enlargement on preoperative CT scans had poorer outcomes than those without chest lesions [18].

Notably, none of the previous studies reported a correlation between pathologically identified SdLNM, which was first detected by PET/CT, and its prognostic significance. Some studies on the detection of SdLNM by preoperative imaging demonstrated that presence of chest lymph node lesions correlated with a poorer prognosis in patients with AEOC [11, 12, 18]. However, one study showed that in cases where PET/CT indicated stage IV disease that was otherwise image stage III by other imaging modalities, the survival of the both groups would be similar [8]. In our study, we performed either node excision or biopsy for 54% of the patients showing SdLNM on PET/CT, which pathologically confirmed metastases. Based on our data, patients with SdLNM only on preoperative PET/CT had worse outcome than those with PET/CT stage III and had a similar progression-free-survival rate as compared to those with PET/CT stage IV with other metastases.

Several procedures were introduced for SdLNM removal in AEOC. VATS has been evaluated as a diagnostic tool for intra-thoracic disease, and is feasible for removal of suspicious lesions in AEOC [19, 20]. In addition, transdiaphragmatic CPLN dissection also was recently introduced as a feasible procedure that does not require the assistance of a thoracic surgeon [21, 22]. However, based on our study data, progression-free-survival of the patients who underwent the supradiaphragmatic procedure during the PDS was not significantly different from that of patients who did not undergo the procedure. In this study, we did not observe a survival benefit for SdLNM resection because of the small numbers of patients who underwent thoracic debulking.

The recurrence pattern in patients with PET/CT stage IV patients could be another factors reasonable for the small benefit of SdLNM removal. Peri et al. reported on the recurrence of stage IV ovarian cancer patients [9]. Similarly, in this study, the recurrence pattern of PET/CT stage IV ovarian cancer was similar to that of PET/CT stage IIIC cancer. Recurrence occurred most frequently in the intra-peritoneal space, and < 10% of the cohort demonstrated thoracic recurrence only. These data may be further evidence to support the fact that supradiaphragmatic procedures confer little benefit to patients with the PET/CT IV with SdLNM.

Our study and other studies question the importance of SdLNM location in AEOC. Previous studies suggested that the anterior chest wall lymph nodes are the major site of SdLNM [8]; however, in this study, SCFLN metastases were the most-prevalent SdLNM identified on PET/CT. It is currently unclear whether SdLNs should be categorized according to their origin of drainage. Hynninen reported [17] that if there are two different lymphatic pathways from the peritoneal cavity to the thoracic cavity, the anterior chest wall lymph node metastases would correlate with PET/CT stage III patients. CPLN might be a thoracic marker of extensive peritoneal stage III disease. Thus, SCFLNs drained from deep pelvic lymph node chains might have a different prognostic impact than CPLN. A redefinition of SdLNM based on location and lymphatic afferents may impact our understanding of residual disease after debulking surgery in AEOC.

Despite our important findings, this study had a limitation. Owing to its retrospective design, there may have been a selection. However, we attempt to recruit a homogenous patient cohort in order to analyze progression-free survival data of patients who underwent PDS. The strength of our study is that SdLNM detected by PET/CT was pathologically confirmed. However, the extent of thoracic debulking varied in this study, which could have affected the progression-free-survival of patients who underwent the supradiaphragmatic procedure.

## Conclusions

Patients with AEOC in whom SdLNM was detected using preoperative PET/CT had a poorer prognosis than those without SdLNM and those with other distant metastases. Removal of SdLN lesions may not affect the survival of patients with AEOC. However, further investigation is warranted to determine whether there are location-dependent survival effects of SdLNM in patients with AEOC.

## Additional file

Additional file 1: **Table S1.** Harzard ratio for recurrence and overall survival. (DOCX 15 kb)

## Abbreviations

AEOC: Advanced epithelial ovarian cancer
CT: Computed tomography
MRI: Magnetic resonance image
PET/CT: Positron emission tomography/computed tomography
SdLN: Supradiaphragmatic lymph nodes
SdLNM: Supradiaphragmatic lymph node metastasis
FIGO: International Federation of Gynecology and Obstetrics
NAC: Neoadjuvant chemotherapy
IDS: Interval debulking surgery
PDS: Primary debulking surgery
VATS: Video-assisted thoracoscopic surgery
SCFLN: Supraclavicular fossa lymph node
AxLN: Axillary lymph nodes
CPNL: Cardiophrenic lymph nodes
