# Supplementary material for: Prognostic significance of supradiaphragmatic lymph node metastasis detected by 18F-FDG PET/CT in advanced epithelial ovarian cancer
Source: BMC Cancer. 2018 Nov 26;18:1165. doi: 10.1186/s12885-018-5067-1 (PMC6260780; doi:10.1186/s12885-018-5067-1)
Supplement: Supplementary file 1 — Table S1. Harzard ratio for recurrence and overall survival. (DOCX 15 kb) [file 12885_2018_5067_MOESM1_ESM.docx]

**Table S1. Harzard ratio for recurrence and overall survival**

| **Variables** |  | **Recurrence HR**  **(95% CI)** | **Death HR**  **(95% CI)** |
| --- | --- | --- | --- |
| **Age** | **≤65** | 1 | 1 |
|  | **>65** | 1.09 (0.71-1.66) | 1.12 (0.58-2.15) |
| **PET/CT stage** | **III** | **1** | 1 |
|  | **IV** | **1.61 (1.13-2.29)** | 1.48 (0.84-2.61) |
| **Histology** | **Serous adenocarcinoma** | 1 | 1 |
|  | **Others** | 0.88 (0.54-1.41) | 0.99 (0.47-2.08) |
| **Grade** | **I** | 1 | 1 |
|  | **II** | 1.66 (0.78-3.53) | 1.02 (0.34-3.10) |
|  | **III** | 1.38 (0.66-2.91) | 1.31 (0.46-3.79) |
| **Optimal resection** | **residual ≤1cm** | **0.52 (0.34-0.81)** | 0.55 (0.29-1.04) |
|  | **residual >1cm** | **1** | 1 |
| HR : Hazard ratio, SdLNM : Supradiaphragmatic lymph node metastasis | | | |
